# Prophylactic influences of prebiotics on gut microbiome and immune response of heat-stressed broiler chickens

**DOI:** 10.1038/s41598-023-40997-7

**Published:** 2023-08-26

**Authors:** Yara Sayed, Mariam Hassan, Heba M. Salem, Khaled Al-Amry, Gamal E. Eid

**Affiliations:** 1 Department of Microbiology, Faculty of Veterinary Medicine, Cairo University, Giza, 12211 Egypt; 2 Department of Microbiology and Immunology, Faculty of Pharmacy, Cairo University, Kasr El-Aini Street, Cairo, 11562 Egypt; 3 Department of Microbiology and Immunology, Faculty of Pharmacy, Galala University, New Galala City, Suez, Egypt; 4 Department of Poultry Diseases, Faculty of Veterinary Medicine, Cairo University, Giza, 12211 Egypt

**Keywords:** Microbial communities, Biodiversity, Climate-change ecology

## Abstract

Climatic changes and elevated ambient temperature are significant environmental stressors with a negative impact on birds’ physiological, immunological, and behavioral status, increasing their susceptibility to stressors and immunosuppression and consequently increasing intestinal permeability (leaky gut). Prebiotics have been utilized to stop or diminish the harmful effects of stress in chickens. We aimed to evaluate the role of mannan-oligosaccharides, and beta-d-glucan prebiotics supplements in drinking water against experimentally induced heat stress (HS) on broiler chickens and study their impact on birds’ performance, gut microbiome, and immune response. A total of 120 1-day-old Ross broiler chicks were allocated into four groups (30 birds/group), and each group was subdivided into triplicates (10 birds each). The experimental groups were classified as follows; the 1st (G1) control birds, the 2nd (G2) birds exposed experimentally to HS, the 3rd (G3) birds administered prebiotics in drinking water without exposure to HS, and the 4th (G4) birds exposed to HS and administered prebiotics in drinking water. After each vaccination, blood samples and serum samples were collected to evaluate the birds’ immune status. Fecal samples were also collected for the molecular evaluation of the gut microbiome based on the genetic analyses and sequencing of 16S rRNA gene. The results showed that HS has reduced the birds’ performance and badly affected the birds’ immune response and gut microbiome. However, the addition of prebiotics to drinking water, with or without stress, enhanced the growth rate, maintained a normal gut microbiome, and improved immune parameters. Moreover, the usage of prebiotics improved the chicken gut microbiome and alleviated the negative effect of heat stress. Administering prebiotics significantly (p < 0.05) increased the relative abundance of beneficial bacteria and eradicated pathogenic ones in the birds’ gut microbiome. Prebiotics showed a positive effect on the gut microbiome and the immune status of chickens under HS in addition to their efficacy as a growth promoter.

## Introduction

The poultry industry in tropical countries is subjected to huge challenges arising from the environmental temperatures that cause heat stress^1^. Heat stress (HS) has become a dangerous issue; it causes huge losses in broiler production and reduces the performance and productivity of broilers^2^. Birds that are exposed to HS show behavioral, structural, and immunological changes that negatively affect the poultry business^3^. The profitability of the broiler production business could be improved by properly managing HS in flocks during the hot season^4^. The increased quantity of eggs and improved quality of chicken meat during the summer months may aid in covering the general public’s protein needs and resolve food deficiency problems in developing nations^5^. In this regard, poultry researchers are working regularly and aggressively to create strategies for producers to combat HS problems in poultry farms. The most conventionally successful method to counteract HS effects on birds is using air cooling/ventilation systems, which have many drawbacks including noise from the operating machines, unequally distributed chickens, and being very expensive to cool the entire housing^6^. Therefore, most approaches applied to mitigate HS have been focused on nutritional approaches^7^. Antibiotics are one of the feed supplements used in the poultry business for treatment, prevention of diseases, and growth development. However, the presence of antibiotic residues in poultry meat harms the health of consumers by causing resistance to the normal microbiota and the dissemination of pathogenic microorganisms as well as, it resulted in the appearance of microbial resistance against antibiotics^8, 9^.

Prebiotics are considered the best feed additives as antibiotic substitutes^9^. Gibson and Roberfroidd^10^ were the first to define a prebiotic as “a non-digestible non-fermentable food element that benefits the host by selectively encouraging the growing and/or activity of one or a restricted number of bacteria in the colon, hence improving host health”^10^. This definition was later revised by Gibson et al. who re-defined a prebiotic as “a selectively fermented food that causes specific changes in the development and/or viability of the intestinal microbiota which improves host health and welfare”^11^. Oligosaccharides have been the most utilized prebiotics in the poultry industry, including mannan oligosaccharides (MOS), inulin, fructo-oligosaccharides (FOS), galacto-oligosaccharides (GOS), xylo-oligosaccharides (XOS), soya-oligosaccharides (SOS), isomalt-oligosaccharides (IMO), pyrodextrins, lactulose, and oligochitosan^12^. The intestinal microbiota can ferment prebiotics to generate short-chain fatty acids (SCFA), lactic acid, or certain antimicrobial substances like bacteriocin against harmful microbial species^13^. The inhibition of harmful microbes in the gut may be attributed to the increased intestinal acidity produced by the fermented products of prebiotics^12^. In turn, prebiotics may help heat-stressed chickens by improving their immune system, gut morphology, oxidative status, and physiological stress responses, which will ultimately improve the growth performance of broiler chickens^2^. Therefore, we aimed to evaluate the mitigating and prophylactic influences of prebiotics on the birds’ performance, gut microbiome, and immune status of heat-stressed chickens.

## Materials and methods

### Birds housing and management

This study was ethically approved by Cairo University Institutional Animal Care and Use Committee (CU-IACUC) Veterinary Medical and Agricultural Sciences Sector with code “CU ll F 27 21”. All the animals procedures in this study were conducted according to the guidelines of the Faculty of Veterinary Medicine Cairo University following the “Guide for the Care and Use of Laboratory Animals” published by the Institute of Laboratory Animal Research (Washington, DC, USA). All the animals procedures conducted in this study are following the ARRIVE guidelines 2.0.

A total of 120, 1-day-old commercial male Ross broiler chicks were obtained from a local commercial hatchery. The chickens were raised in the experimental station of the Faculty of Veterinary Medicine, Cairo University during February and March 2022. The pens were disinfected by TH4 (a combination of glutaraldehyde and quaternary ammonium compounds, Sogeval, France) before housing. The poultry house was cleaned, and all manure materials were removed then, water and high-pressure sprayers were used to remove dust materials from windows, floors, ceilings, walls, and ventilation shafts, which were subsequently disinfected with TH4 that had been diluted to 1:200. All removable fittings and equipment (feeders and drinkers) were removed from the building and submerged in 1:200 TH4 dilution baths. Quicklime was sprayed on the ground to destroy parasites and their eggs, such as coccidia. Sixty-watt bulb was hung 2.2 m in the middle of each pen. Light intensity at the level of the birds was approximately 2.66 lx/m^2^/s^14^. Dark/light cycle of the broilers during the experiment are demonstrated in Table 1. Chicks were allocated into four groups, each group included 30 chicks. Each group was subdivided into 3 replicates (10 birds each). Chickens’ subgroups were raised in open-side poultry house, and kept on a deep litter system, in separate pens with coarse sawdust bedding material with 10 cm thickness on a concrete floor. The litter was maintained and the wet parts around the drinkers were replaced with dry ones. A good ventilation system was applied to provide continuous airflow and prevent the accumulation of harmful gases such as carbon dioxide and ammonia via air vents, windows, as well as mechanical ventilation by fans and air suction. The birds were administered a balanced commercial diet without any feed additives and administered drinking water (clean and clear tap water, pH 7, room temperature) ad libitum.

**Table 1:** Dark/light cycle of the broilers during the experiment.

Time	Duration of dark (h)	Duration of light (h)
First 3 days	0	24
1st week	1	23
2nd week	2	22
3rd week	3	21
4th and 5th week	4	20

### Temperature and humidity of the poultry house

At the beginning of chicks’ rearing, the temperature was set at 35 °C and steadily decreased at a pace of 2–5 °C/week until it reached 22 °C at the end of the 4th week and then the temperature was kept at 22 °C for the 5th week^15^. The maximum and minimum thermometer (Taylor Precision 5154 Wall Thermometer Cat# 5154) readings were taken every day to determine the ambient temperature. At weeks 4 and 5, the temperature was kept at 32–35 °C for the two heat-stressed groups using an electrical heater. A wall-mounted thermo-hygrometer (Oumar Hygrothermograph, Cat# TH108) was used to detect indoor relative humidity where the average relative humidity ranged from 60 to 70%. According to the formula described by Farghly et al.^1^, the average temperature humidity index (THI), was 31.427.

### Feeding, and vaccination program for the experimental chickens

All the birds were fed a commercial balanced ration without any feed additives (Cairo Poultry company). Chicks were fed a starter diet for the first 13 days, a grower from 14 to 26 days old, and a finisher diet from 27 to 35 days of age. The percentage of proteins, fats and fibers in the starter, grower and finisher rations are demonstrated in Table 2. The composition of the starter ration was as follow: yellow maize, soybean gain (46%), high fat extruded soybean, yellow corn gluten (60%), DDGS, monocalcium phosphate, limestone powder, common salt, dl-Methionine, and l-lysine hydrochloride, a mixture of vitamin and mineral salt (1/8219) choline chloride. The grower ration composition was as follow: yellow maize, soybean gain (46%), high fat extruded soybean, yellow corn gluten (60%), vegetable oils (cotton seed, sunflower, soy oil), monocalcium phosphate, limestone powder, common salt, dl-Methionine, and l-lysine hydrochloride, a mixture of vitamin and mineral salt (1/8219) choline chloride. The finisher ration composition was as follow: yellow maize, soybean gain (46%), high fat extruded soybean, yellow corn gluten (60%), gluten feed (22%), monocalcium phosphate, limestone powder, common salt, dl-Methionine, and l-lysine hydrochloride, a mixture of vitamin and mineral salt (1/8219) choline chloride. Each ton of this ration contains the nutritional needs of vitamins and minerals in internationally recommended proportions. Vitamins included A, D3, E, B1, B2, B6, B12, niacin, biotin, folic Acid, choline chloride, and pantothenic acid. Mineral elements included manganese, iodine, iron, copper, cobalt, selenium, and zinc. During the first 12 h post-arrival, chicks were supplied with rehydration salts-treated water (glucose anhydrous 2.70 g, trisodium citrate anhydrous 0.58 g, sodium chloride 0.52 g, and potassium chloride 0.30 g for each sachet (4.1 g) of the product. During the experiment, a vaccination program was adopted as follows (Table 3); on day one, all birds were vaccinated against Newcastle disease (ND) and Infectious Bronchitis (IB) using Nobilis Ma5 + Clone 30 live vaccine, via spraying method. On day 7, birds were vaccinated via subcutaneous injection with 0.5 mL at the base of the neck against avian influenza H5N1 using Egymune H5 inactivated reassortant vaccine. The birds were also vaccinated against Gumboro (Infectious Bursal Disease) using Nobilis GUMBORO D78 live vaccine through drinking water on day 12. Finally, the birds were vaccinated again against ND by Nobilis, LaSota strain live vaccine in drinking water on day 17.

**Table 2:** The composition of ration used during the experiment.

	Starter	Grower	Finisher
Crude protein (%)	23	21	18
Crude fat (%)	5.85	7.14	6.70
Metabolizable energy (kcal/kg)	3050	3150	3200
Crude fiber (%)	3.50	3.49	3.65

**Table 3:** Details of the vaccine program adopted during the experiment.

Vaccine	Disease	Type	Concentration	Method	Time
Nobilis Ma5 + Clone 30	Infectious bronchitis (IB) and Newcastle disease (ND)	Live vaccine	Live I.B. strain Ma5: ≥ 3 log_10_ EID_50_ Live ND strain Clone 30: ≥ 6 log_10_ EID_50_	Spray	Day 1
Egymune H5	Avian influenza H5N1	Inactivated reassortant vaccine	8 log_10_ EID_50_/mL	Subcutaneous injection	Day 7
Nobilis GUMBORO D78	Infectious bursal disease	Live vaccine	≥ 4 log_10_ TCID_50_	Drinking water	Day 12
Nobilis LaSota strain	Newcastle disease (ND)	Live vaccine	≥ 6 log_10_ EID_50_	Drinking water	Day 17

*EID*_*50*_ Median embryo infectious dose, *TCID*_*50*_ median tissue culture infectious dose.

### Prebiotics constitutions and dose

Commercial prebiotic Hydrostar from EGYEURO Company (Cat# 32295000) was applied in drinking water at a dose of 0.5 mL/L (following the manufacturer's instructions) throughout the rearing period from day one till the end of the experiment. The constitutions of the prebiotic Hydrostar are shown in Table 4.

**Table 4:** Hydrostar prebiotics (liquid yeast hydrolysate) composition.

Crude protein, minimum	6%
Crude fat, minimum	1%
Crude fiber, minimum	2.5%
Cerevisiae yeast	222.222 mg/L
Mannan	8880 mg/L
Glucan	13.330 mg/L
Xanthan gum	11.111 mg/L
Acetic acid	11.111 mg/L
Coloring (E-133)	11 mg/L

### Experimental design

As demonstrated in Table 5, this study was conducted using four groups with two main factors: heat stress (32–35 °C) and the administration of prebiotics. The 1st group (G1) was the control negative group, the 2nd group (G2) birds were exposed to experimentally HS, the 3rd group (G3) was administered prebiotics in drinking water without exposure to HS, and the 4th group (G4) was exposed to HS and administered prebiotics in drinking water. The addition of the prebiotics started on day 1 and until the end of the experiment while HS exposure began at week 4 till the end of the experiment.

**Table 5:** Experimental design; prebiotics treatment with or without heat stress of the chicken study groups.

Factor	Groups			
	Control group (G1)	Heat stress group (G2)	Prebiotic group (G3)	Heat stress + prebiotic (G4)
Prebiotics	Absent	Absent	Present	Present
Temperature degree	Ambient temperature (22 °C)	High temperature (32–35 °C)	Ambient temperature (22 °C)	High temperature (32–35 °C)

#### Body weight (BW), weight gain (WG), and cumulative mortality

Chicks were individually weighed at 1-day old (starting BW) and weekly throughout the experimental period (1–35 d). The live body weight gain (WG) was calculated weekly by subtracting the initial weight from the current weight and expressed as (g/bird/week). The cumulative mortality rate was calculated as the total number of deaths in chickens per group divided by the total population in the same group.

#### Feed intake (FI) and Feed conversion ratio (FCR)

Each group of chicks received a set amount of feed weekly, and the remaining feed was weighed at the end of each week. The difference between the feed delivered and the rest of the feed was estimated. The weekly consumed feed was divided by the number of birds in each group this week to get the average quantity of feed consumption, so it was expressed as (g/bird/week). Regarding the feed conversion ratio (FCR), it was calculated weekly by dividing the average feed consumption per bird by the average body weight gain per bird.

### Sampling

#### Blood samples

Nine birds were randomly selected weekly from each group and 4.5 mL (0.5 mL per bird) of blood samples were ethically collected from the wing vein into 2 tubes (with and without anticoagulant). The blood samples without anticoagulant were cooled to approximately 4 °C overnight then centrifuged at 3000 rpm for 10 min and sera were transferred to clean tubes using a micropipette (Eppendorf Research Plus micropipette, adjustable volume 20–200 µL). The collected sera were kept at − 20 °C, until the analysis of the hemagglutination inhibition (HI)^14^. Meanwhile, blood samples taken into tubes with EDTA as an anticoagulant were used for immune cells counting (total and differential leukocytes).

#### Fecal samples for microbiome analysis

Total genomic DNA was extracted from the fecal sample (200 mg, three samples from each group, a total of 600 mg per group) at three-time intervals (1st, 3rd, and 5th week) using the commercially available QIAamp Fast DNA Stool Mini kit (QIAGEN, Germany), following the manufacturer’s instructions. DNA concentration and quality were assessed using a nanophotometer (NanoPhotometer P360, Implen) and agarose gel electrophoresis. DNA was stored at − 20 °C until further processing. Table 6 illustrates pooled samples from the same group and sample characterization (sampling time, with/without exposure to HS and prebiotics treatment).

**Table 6:** Fecal DNA samples for gut microbiome analysis and characterization at exposure to prebiotics with or without heat stress.

Sample code	Number of pooled samples	Time of sample	Heat stress	Prebiotics
S1	4 Samples represent all groups before the treatment	Day 1 (Week 1)	No	No
S2	3 Samples of the control group	Week 3	No	No
S3	3 Samples of the prebiotics group	Week 3	No	Yes
S4	3 Samples of the control group	Week 5	No	No
S5	3 Samples of the prebiotics group	Week 5	No	Yes
S6	3 Samples of the heat stress group	Week 5	Yes	No
S7	3 Samples of the heat stress + prebiotics group	Week 5	Yes	Yes

### DNA sequencing and gut microbiome data analysis

Sequencing using Illumina-Miseq™ (2 × 300 bp paired-end protocol) was performed targeting the bacterial V3 and V4 regions of the 16S rRNA gene^16^. Generated Illumina reads were analyzed using QIIME tool version 1.9.1^17^ as described before^18, 19^.

### Total and differential leukocytic counts

The whole blood samples were used for total and differential leukocytic counts. The total count was carried out microscopically using a hemocytometer (Standard Hemocytometer Weberscientific Cat# 3048-12) after mixing 1 mL (Eppendorf Research Plus micropipette, adjustable volume 100–1000 µL) of Natt and Herrick solution with 10 µL (Eppendorf Research Plus micropipette, adjustable volume 0.5–10 µL) blood and was examined under microscope (Binocular optical microscope Celestron labs) using a hemocytometer. The differential count was done using the commercial Diff Quick stain^20^.

### Antibody (HI) titers to ND and AI vaccines

The hemagglutination inhibition (HI) test was applied to measure the serum level of HI antibodies to the given Newcastle disease (ND) and avian influenza (AI) vaccines. Briefly, two-fold serial dilutions of the sera were mixed with an equal volume of adjusted viral HA antigens. Chicken red blood cells (1% RBCs, v/v) were added, and the end point was indicated by the highest dilution that inhibited the agglutination of the chicken RBCs by observing the button formation rather than agglutination^21^.

### Statistical analysis

Values were expressed as mean ± stranded error (M ± S.E). Statistical comparisons among the means of different experimental groups were constructed with t-test in weeks 1, 2 and 3, and one-way ANOVA in weeks 4 & 5 in all tests. All tests were made by GraphPad Prism version 9.0.2 (GraphPad Software, San Diego, California USA, www.graphpad.com). A probability “*P*” value of ≤ 0.05 was considered statistically significant.

For the microbiome samples we used PERMANOVA (permutational multivariate analysis of variance) and ANOSIM (analysis of similarities) using Python script embedded in QIIME^17, 19^ (1.9.1); these tests were used for the analysis of the strength and the statistical significance of sample groupings. We did the differential abundance analysis aiming to find the differences in the relative abundance of each taxa between samples and then we used statistical analysis to assign a significant value to each comparison. We used Kruskal–Wallis and Bonferroni’s multiple comparisons tests using Python scripts embedded in QIIME^17, 19^ (1.9.1), for each taxa the* P* value and the false positive adjustment of the *P* value (FDR P value) corrected by the Benjamini–Hochberg FDR procedure for multiple comparisons were calculated.

## Results

### Body weight (BW), weight gain (WG), and cumulative mortality

As shown in Table 7, HS reduced the BW of broilers in the 4th and 5th weeks in G2. In the 4th week, birds in G1 showed a higher BW (1831 ± 15.46 g) compared to birds exposed to HS in G2 (1763 ± 18.25 g). The administration of prebiotics to HS-exposed broilers (G3) counteracted the average BW loss in the same week when compared to groups without heat stress. However, in the 5th week, the average BWs of G1 and G2 were 2450 g and 2250 g, respectively while the average BWs of birds in G3 and G4 were 2340 g and 2287 g, respectively.

**Table 7:** Impacts of prebiotics with or without heat stress on the body weight, weight gain, feed intake, and feed conversion ratio (FCR) of broilers.

Time	Groups	Body weight (g)	Weight gain (g)	Feed intake (g)	FCR
Week 1	Control	185 ± 2.88^a^	146 ± 2.88^a^	163.3 ± 12.81	1.117 ± 0.06
	Prebiotics	187 ± 2.79^b^	148 ± 2.79^b^	159.2 ± 9.33	1.074 ± 0.04
Week 2	Control	523 ± 9.66^a^	338 ± 6.77^a^	420.5 ± 18.58	1.243 ± 0.03^a^
	Prebiotics	534 ± 8.59^b^	347 ± 5.79^b^	426.4 ± 16.81	1.228 ± 0.02^b^
Week 3	Control	1089 ± 12.88^a^	566 ± 3.21^a^	734.3 ± 34.42^a^	1.297 ± 0.05^a^
	Prebiotics	1064 ± 12.03^b^	530 ± 3.43^b^	682.4 ± 32.3^b^	1.287 ± 0.05^b^
Week 4	Control	1831 ± 15.46	742 ± 2.57^a^	1047 ± 51.39	1.41 ± 0.06
	Prebiotics	1785 ± 15.03	721 ± 3.00^b^	1049 ± 58.6	1.455 ± 0.07
	Heat stress	1763 ± 18.25	674 ± 5.36^c^	949 ± 65.53	1.407 ± 0.08
	Heat stress prebiotics	1759 ± 10.74	695 ± 1.28^d^	1008 ± 51.67	1.45 ± 0.07
Week 5	Control	2450 ± 19.05^a^	619 ± 4.04^a^	1278 ± 61.2	2.063 ± 0.08
	Prebiotics	2340 ± 12.7^b^	555 ± 2.3^b^	1126 ± 31.47	2.029 ± 0.06
	Heat stress	2250 ± 23.62^c^	487 ± 5.36^c^	1121 ± 64.63	2.300 ± 0.1
	Heat stress prebiotics	2287 ± 17.18^b,c^	527.8 ± 6.64^d^	1064 ± 52.65	2.013 ± 0.07
Final (cumulative day 1–35)	Control	–	2411.00 ± 19.06^a^	3642 ± 178.1	1.51 ± 0.06
	Prebiotics	–	2301.00 ± 21.36^b^	3443 ± 148.4	1.496 ± 0.05
	Heat stress	–	2211.00 ± 23.67^c^	3389 ± 196	1.531 ± 0.07
	Heat stress prebiotics	–	2260.00 ± 21.07^b,c^	3340 ± 163.1	1.485 ± 0.06

Each value represents mean ± S.E. Significant difference between groups in body weight, weight gain, feed intake, and FCR was analyzed by t-test in weeks 1, 2 and 3, and one-way ANOVA in weeks 4 and 5 at p ≤ 0.05.

Parameters in the same column followed by different superscript letters are significantly different (p ≤ 0.05).

During the 4th week, the WG in the birds in G1 and G2 were 742 g and 674 g, respectively while in G3 and G4 were 721 g and 695 g, respectively. During the 5th week, the WG in the birds in G1 and G2 were 619 g and 487 g, respectively while, in G3 and G4 they were 555 g and 527 g, respectively. Final WG in the birds in G1 and G2 were 2411 g and 2211 g, respectively while, in G3 and 4, they were 2301 g and 2260 g, respectively.

There was a significant increase in BW of G3 than G1 in the first 2 weeks (*p* = 0.0023), (p = 0.0094) respectively, and a significant decrease in BW in the same group (*p* = 0.0012) in week 3. Meanwhile, in the 4th week, there was a significant increase between G1 and G4 (*p* = 0.0396). In the 5th week, there was a significant increase between G1 and G4, G1 and G3, G1 and G2, and G3 and G2 (*p* = 0.0012), (*p* = 0.0129), (*p* = 0.0003), (*p* = 0.0364) respectively.

Concerning the WG, there was a significant increase between G3 and G1 in the first 2 weeks (*p* = 0.0023) and (*p* = 0.0116) respectively, in contrast, a significant decrease in WG in the same groups (*p* ≤ 0.0001) in week 3. While in week 4, there was a significant increase among all groups G1 & G4, G1 & G2, G3 & G2 (*p* ≤ 0.0001), and a significant increase in G1 & G3, G3 & G4 (*p* = 0.0102) and (*p* = 0.0028) respectively. Finally, there was a significant decrease in G2 & G4 (*p* = 0.0102).

In week 5, there were a significant increase between G1 & G4, G1 & G3, G1 & G2, G3 & G2 (*p* < 0.0001), while a significant increase between G3 & G4 (*p* = 0.0176), and a significant decrease between G2 & G4 (*p* = 0.0016).

Finally, WG (1–35) shows a significant increase between G1 & G3, G1 & G2, and G1 & G4 (*p* = 0.0271), (*p* = 0.0008) and (*p* = 0.0046), respectively. The cumulative mortalities have been recorded to be at the highest level of 10% (3/30 birds) in G2 (HS group) followed by G1, and G4 (control group and heat stress prebiotics group) with a rate of 3% (1/30 birds) for each group while no mortalities were recorded in G3 (prebiotics group) all over the experimental period.

### Feed intake (FI) and Feed conversion ratio (FCR)

Birds in G2 and G4 showed a mathematically reduced FI than those in G1 and G3. The FI masses of chickens in the 4th week in G1 and G3 were 1047 g and 1049 g, respectively, while in G2 and G4 the FI values were 949 g and 1008 g, respectively. In the 5th week, the FI of chickens in G1 and G3 were 1278 g and 1126 g, respectively, while in G2 and G4 they were 1121 g and 1064 g, respectively. Unexpectedly, the administration of prebiotics to chickens of the control group led to a drastic decline in the FI while the decline was not that much in heat-stressed chickens. The addition of prebiotics to heat-treated birds did not cause a severe decline in FI when compared to the control group of the same week which caused a great decrease in FI.

In week 4, G1 & G2 were 1047 and 949, while G3 & G4 were1049 and 1008, respectively. At week 5, G1 & G2 were 1278 and 1121, while G3 & G4 were 1126 and 1064, respectively.

The overall FI masses in G1 & G2 were 3642 and 3389, while in G3& G4 they were 3443 g and 3340, respectively, and there was a significant increase in FI only in week 3 (*p* = 0.0017) when G1 compared with G3. In the 4th week, the FCR in the chickens of G2 and G4 were 1.40 and 1.45, respectively while G1 and G3 showed FCR values of 1.41 and 1.45, respectively. In the 5th week, the FCRs in G2, G4, G1, and G3 were 2.30, 2.01, 2.06, and 2.02, respectively. The overall FCRs were 1.53, 1.48, 1.51, and 1.49, in the same group order respectively. The overall FCRs in prebiotics-treated groups were better than those in other groups. There was a significant increase between G1, and G3, in week 2 & 3 (*p* = 0.0229) and (*p* = 0.0131), respectively. Table 4 depicts the BW, WG, and FCR of broilers as related to prebiotic administration and heat stress.

### Total and differential leukocytic counts

Data concerning the total white blood cells count (TWBC) X 10^3^/mm^3^ were presented by means ± standard errors (Mean ± S.E.) as summarized in Table 8. A significant difference was noticed between the TWBC of chickens of different groups in week 4. The corresponding *p* values with significant increase were (*p* ≤ 0.0001) for G1 vs G3, G1 vs G2, G1 vs G4, G3 vs G2, significant increase (*p* = 0.0011) for G3 vs G4, and significant decrease (*p* = 0.0212) for G2 vs G4. At week 5, there was a significant increase between G1 when compared with G3, and G1 with G4 (*p* ≤ 0.0001), G1 with G2 (*p* = 0.0024), G3 with G4 (*p* = 0.0191), and G2 with G4 (*p* = 0.0007).

**Table 8:** Effects of prebiotics with or without heat stress, and their interaction on total and differential leukocyte count.

Time	Groups	WBC × 10^3^ cells/mm^3^	Lymphocyte%	Monocyte%	Heterophil%	Eosinophil%
Week 2	Control	72.78 ± 4.573	48.444 ± 0.930^a^	12.111 ± 1.467^a^	38.444 ± 1.684	1.000 ± 0.577
	Prebiotics	81.11 ± 4.47	37.333 ± 1.658^b^	27.333 ± 2.048^b^	35.333 ± 0.408	0.000 ± 0.000
Week 3	Control	67.22 ± 3.84	58.000 ± 1.247	17.778 ± 0.909^a^	22.667 ± 1.054	1.556 ± 0.294
	Prebiotics	65.78 ± 1.47	55.778 ± 1.024	23.556 ± 1.144^b^	20.667 ± 1.915	0.000 ± 000
Week 4	Control	83.33 ± 1.78^a^	58.667 ± 0.667^a^	21.111 ± 0.351^a^	19.111 ± 0.588^a^	1.111 ± 0.351
	Prebiotics	70.33 ± 0.781^b^	61.111 ± 0.351^b^	21.333 ± 1.106^a^	17.556 ± 1.281^a^	0.000 ± 0.000
	Heat stress	56.57 ± 0.781^c^	62.444 ± 1.281^c^	29.111 ± 0.676^b^	8.444 ± 0.930^b^	0.000 ± 0.000
	Heat stress prebiotics	62.44 ± 1.634^d^	62.667 ± 0.745^b,c^	26.889 ± 0.351^b,c^	10.000 ± 0.471^b^	0.444 ± 0.294
Week 5	Control	91.56 ± 3.245^a^	44.667 ± 1.054	16.667 ± 1.106^a^	35.333 ± 1.054^a^	3.333 ± 0.333
	Prebiotics	74.22 ± 3.022^b^	45.333 ± 0.667	23.444 ± 1.119^b^	29.222 ± 1.451^b^	2 ± 0.333
	Heat stress	78.44 ± 0.987^c^	42 ± 0.969^a^	40.889 ± 1.338^c^	16.444 ± 1.281^c^	0.444 ± 0.294
	Heat stress prebiotics	63.78 ± 1.341^d^	46.444 ± 0.801^b^	34.222 ± 1.47^d^	18.444 ± 1.281^c^	0.889 ± 0.351

Each value represents mean ± S.E. Significant difference between groups in total WBCS count and differential leukocytic counts was analyzed by t-test in weeks 2 and 3, and one-way ANOVA in weeks 4 and 5 at p ≤ 0.05.

Parameters in the same column followed by different superscript letters are significantly different (p ≤ 0.05).

Data concerning differential leukocytic counts are summarized also in Table 8 which shows that lymphocyte % decreased significantly, and monocyte % increased significantly (*p* ≤ 0.0001) in week 2 when the prebiotics-treated birds are compared to control groups. In week 3, the monocyte% showed a significant increase (*p* = 0.0004). In week 4 lymphocyte% shows a significant decrease (*p* = 0.0002), (*p* = 0.0407) and (*p* ≤ 0. 0001) in G1 vs G2, G1 vs G3, and G1 vs G4 respectively, while in monocyte% show a significant decrease in G1 when compared with G2, G1 with G4, G3 with G2, and G3 with G4 (*p* ≤ 0.0001), also heterophil showed a significant increase between G1 & G2, G1 & G4, G3 & G2 and G3& G4 (*p* ≤ 0.0001). In week 5, lymphocyte % show a significant decrease (*p* = 0.0048) in G2 with G4, while monocyte % show a significant decrease in G1 when compared with G3, G1 with G2, G1 with G4, G3 with G2, G3 with G4, and significantly increase when G2 compared with G4 at *p* value (*p* ≤ 0. 0001), but heterophil % shows a significant increase in G1 when compared with G3, G1 with G2, G1 with G4, G3 with G2, and G3 with G4 (*p* ≤ 0.0001).

### HI antibody titers against Newcastle disease and Avian Influenza vaccines

As titrated by the hemagglutination inhibition (HI) test, levels of HI antibodies (log 2) against ND & AI vaccines are presented by mean ± S.E. in Table 9. In week 3, there was a significant decrease in antibody titers when G1 compared with G3 against Al (*p* = 0.0007). In week 4, there was a significant decrease against ND when G1 compared with G3 (*p* = 0.0017), G1 compared with G4 (*p* = 0.0332), and G2 compared with G4 (*p* = 0.0017). Finally, there was a significant increase when G3 compared with G2 (*p* ≤ 0.0001).

**Table 9:** Impact of prebiotics with or without heat stress on hemagglutination inhibition (HI) antibody titers against Newcastle disease (ND) and avian influenza (AI) vaccines.

Time	Groups			
	Control HI titers (Log2)	Prebiotics HI titers (Log2)	Heat stress HI titers (Log2)	Heat stress prebiotics HI titers (Log2)
Week 2 (ND)	2.22 ± 0.147	2.667 ± 0.166	**–**	**–**
Week 3 (Al)	3.778 ± 0.277^a^	5.333 ± 0.235^b^	**–**	**–**
Week 4 (ND)	1 ± 0^a^	1.778 ± 0.147^b^	0.777 ± 0.147^a,c^	1.556 ± 0.175^b,d^
Week 5 (ND)	1.222 ± 0.147	1.444 ± 0.175	0.888 ± 0.2	1.444 ± 0.175

Each value represents mean ± S.E. A significant difference between groups was analyzed by t-test in weeks 2 and 3, and one-way ANOVA in weeks 4 and 5 at p ≤ 0.05. “–” means that the test was not performed as the heat stress started from week 4.

Parameters in the same row followed by different superscript letters are significantly different (p ≤ 0.05).

### Chicken gut microbiome analysis

The high-throughput Illumina-MiSeq sequencing was used to investigate the dynamics of gut microbiota in the investigated chicken groups. A total of 22 samples were pooled into 7 samples (Table 6) that were sequenced and the sequencing output was analyzed. The sequenced samples represented different time intervals of the experiment and the four chicken groups investigated. Sequencing of the 7 fecal samples yielded a total of 888,081 16S rRNA gene sequence reads with an average length of 460 bp after the sequence quality-filtering step. The number of sequences per sample ranged from 189,884 to 97,647 with an average of 126,868.714 ± 27,880.175. The number of observed OTUs per sample ranged from 5206 to 2280 with an average of 3521 ± 934.626. The Good’s coverage of the sequenced samples ranged from 98.08 to 99.29%.

**Figure 1:**
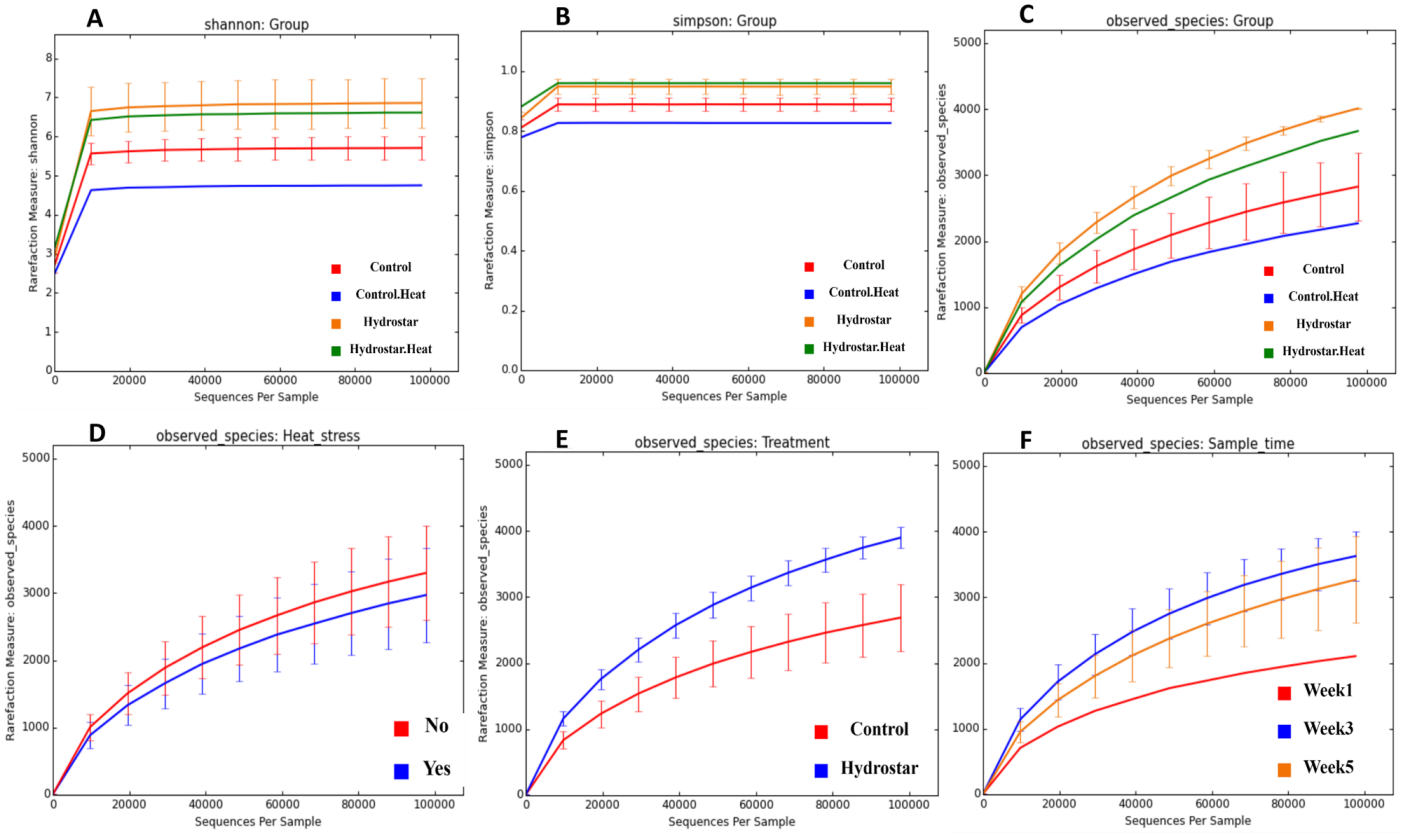
Rarefaction curves of alpha diversity metrics assessed for the sequenced fecal samples. (**A**) Shannon index representing the diversity within samples. (**B**) Simpson’s index (similarity index) revealing species’ evenness. (**C–F**) The observed species representing the true species richness within different sampling groups (Heat stress, Prebiotics treatment (Hydrostar) and Sample time, respectively).

The alpha diversity in the four tested groups was calculated and evaluated. The species diversity was investigated using Simpson’s and Shannon’s metrics (Fig. 1A,B). There was a significant difference recorded between the prebiotics-treated groups and the other groups in terms of Simpson’s and Shannon’s metrics (Monte Carlo permutations (999), (*p* = 0.035 and 0.056), respectively. The number of species observed was the highest in G3 followed by G4, G1 then G2 (Fig. 1C). There was no significant effect of the HS on the number of observed species within samples that were treated with prebiotics when compared to the control samples (Monte Carlo permutations (999), (*p* = 0.689) (Fig. 1D). However, treatment with prebiotics significantly increased the number of observed species within samples (Monte Carlo permutations (999), (*p* = 0.054) (Fig. 1E). Regarding the sampling time, the least number of observed species was during week 1 followed by week 5 and then week 3 with no recorded significant difference (Monte Carlo permutations (999), (*p* = 0.963) (Fig. 1F).

**Figure 2:**
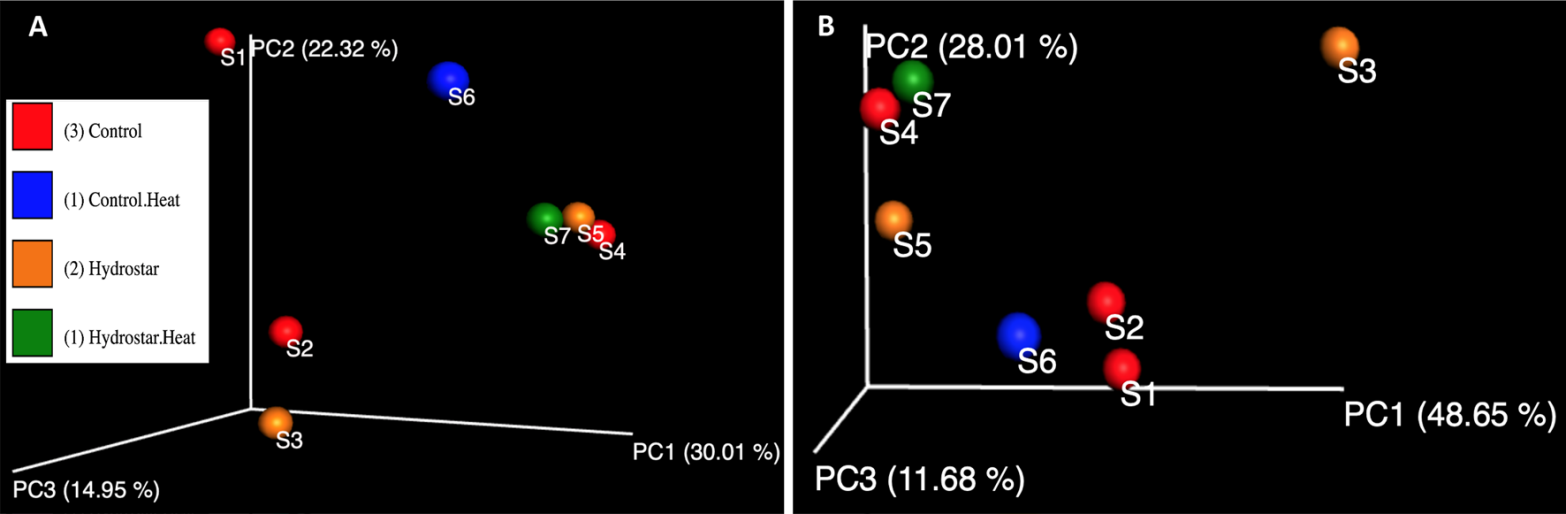
Principal coordinates analysis (PCoA) demonstrating the beta diversity evaluated by the unweighted UNIFRAC (**A**) and weighted UNIFRAC (**B**) method. Individual datasets are designated as spheres which are colored in relation to their sample treatment as follows; red: control (no heat stress or prebiotics), blue: control under heat stress, orange: prebiotics (hydrostar®), and Green (dark): prebiotic under heat stress.

The unweighted and weighted beta diversity between G1, G2, G3, and G4 samples was investigated using the Unifrac distance metrics (Fig. 2A,B). Based on the principal coordinates analysis plot (PCoA), all the prebiotics samples in week 5 (with and without HS) clustered together away from the HS sample (S6) in the same week (Fig. 2A,B). The impact of the prebiotics administration and HS application on the overall microbial community diversity between samples was investigated. There was a significant effect of prebiotics administration on the overall microbial community composition between individual samples (PERMANOVA, *p* = 0.058). The application of HS was accompanied by a significant effect on the overall microbial community composition between individual samples (PERMANOVA, *p* = 0.017).

The effect of the sampling time on the microbial community composition was analyzed. The microbial populations of week 1, week 3, and week 5 were compared to test the significance of sampling time on the total microbial diversity. There was a significant effect of sampling time on the overall community composition between individual samples (PERMANOVA, *p* = 0.008 and ANOSIM, *p* = 0.004).

**Figure 3:**
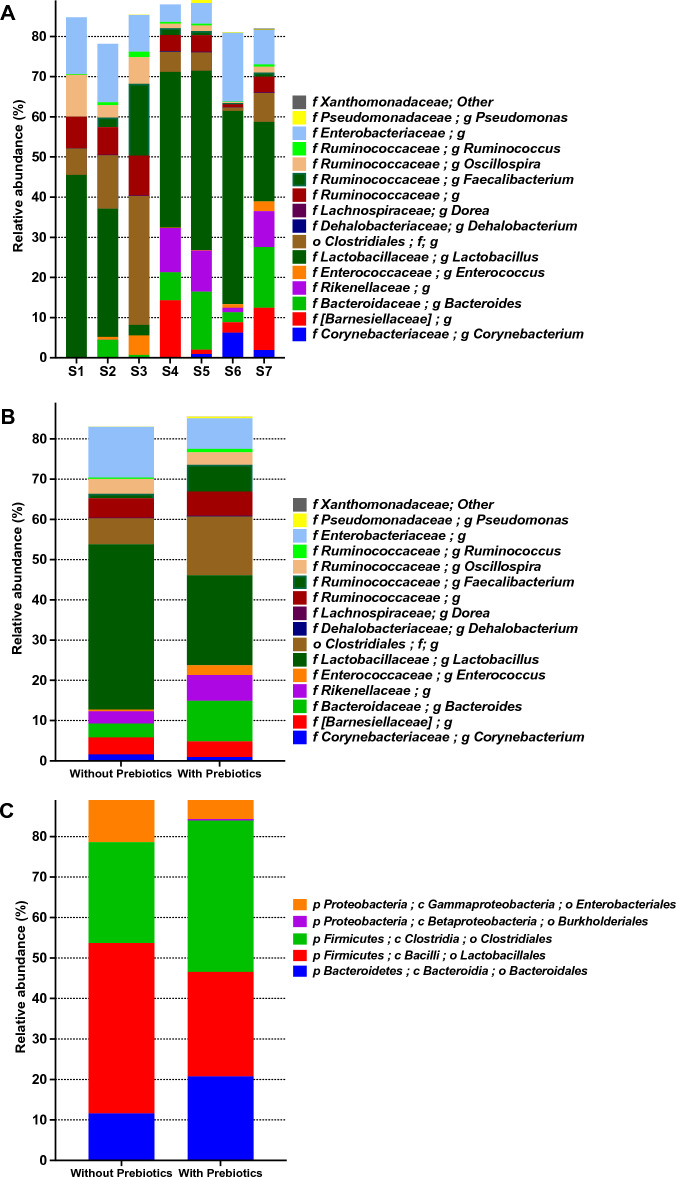
The relative abundance of the most dominant categories of bacteria in prebiotics-treated samples. (**A**) The relative abundance of the most dominant bacteria genera in the different samples. S1 represents all groups before any treatment on day zero. S2 and S4 represent the control group (without prebiotics treatment and without heat stress) in Week 3 and week 5, respectively. S3 and S5 represent the prebiotics group in Week 3 and week 5, respectively. S6 represents the heat stress group in week 5. S7 represents the heat stress + prebiotics group in week 5. (**B**) The relative abundance of the most dominant genera in prebiotics-treated and non-prebiotics-treated samples. (**C**) The relative abundance of the most dominant order in prebiotics-treated and non-prebiotics-treated samples.

**Figure 4:**
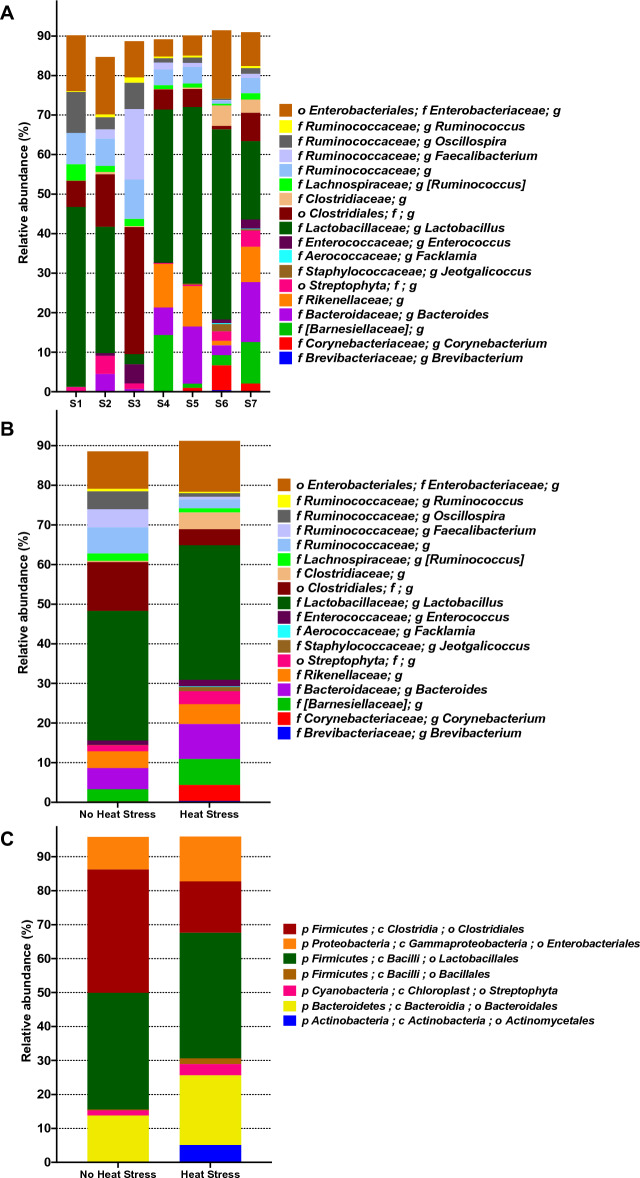
The relative abundance of the most dominant bacteria in heat-stressed samples. (**A**) The relative abundance of the most dominant bacterial genera in the different samples. S1 represents all groups before any treatment on day zero. S2 and S4 represent the control group (without prebiotics treatment and without heat stress) in week 3 and week 5, respectively. S3 and S5 represent the prebiotics group in week 3 and week 5, respectively. S6 represents the heat stress group in week 5. S7 represents the heat stress + prebiotics group in week 5. (**B**) The relative abundance of the most dominant genera in heat-stressed and non-heat-stressed samples. (**C**) The relative abundance of the most dominant orders in heat-stressed and non-heat-stressed samples.

The composition of the microbiota was further examined (Figs. 3, 4). In the prebiotics-treated samples, the major components at the genus level belonged to *Lactobacillus* (22.3%) (Fig. 3A,B). In addition, undefined genera of the order *Clostridiales* (14.6%), family *Bacteroidaceae* (10.0%), and family *Enterobacteriaceae* (7.6%) were detected. On the contrary, in samples whose chickens were not treated by prebiotics the relative abundance of those genera was (41.0%), (6.4%), (3.4%) and (12.5%), respectively, with no significant differences (Kruskal–Wallis, Bonferroni’s multiple comparisons tests, (*p* > 0.1) (Fig. 3B, Table S1). However, there was a significant difference in other bacterial categories as *Pseudomonadaceae*, *Dorea,* an undefined genus of order *Lactobacillales, Dehalobacterium,* and an undefined genus of family *Xanthomonadaceae* (*p* = 0.077) with relative abundance in prebiotics-treated and non-prebiotics-treated samples of (0.34% and 0.02%), (0.133% and 0.067%), (0.06% and 0.011%), (0.0147% and 0.0024%) and (0.06% and 0%) respectively (Fig. 3B, Table S1). Figure 3C describes the relative abundance of the most dominant orders in prebiotics-treated and non-prebiotics-treated samples as follows: *Enterobacteriales* (7.73% and 12.81%), *Burkholderiales* (0.36% and 0.02%), *Clostridiales* (37.39% and 24.91%), *Lactobacillales* (25.80% and 42.09%) and *Bacteroidales* (20.76% and 11.61%), respectively.

The major bacterial taxa and their relative abundance in the fecal samples in both heat-stressed and non-stressed groups were examined (Fig. 4A,B). In the heat-stressed groups, the major components at the genus level were belonging to *Lactobacillus* (33.9%), undefined genus of family *Enterobacteriaceae* (12.9%), *Bacteroides* (8.8%), undefined genus of family *Barnesiellaceae* (6.5%), undefined genus of family *Rikenellaceae* (5.0%), an undefined genus of family *Clostridiaceae* (4.0%), an unknown genus of order *Streptophyta* (3.2%), *Enterococcus* (1.6%), and *Ruminococcus* (1.0%) (Fig. 4B). Concerning the non-stressed bird samples, the relative abundances of those genera were (32.7%), (9.4%), (5.3%), (3.0%), (4.2%), (12.3%), (1.5%), (1.1%) and (1.9%), respectively, with no significant differences (Kruskal–Wallis, Bonferroni’s multiple comparisons tests, p > 0.1) (Fig. 4B, Table S2). However, there was a significant difference in other bacterial genera as an undefined genus of family *Clostridiaceae*, *Corynebacterium, Jeotgalicoccus*, and *Brevibacterium* (*p* = 0.05) with relative abundance in heat-stressed and non-stressed samples (4.25% and 0.25%), (4.09% and 0.19%), (1.08% and 0.04%), and (0.2718% and 0.0076%) respectively (Table S2). In addition to genus *Facklamia* (*p* = 0.04) with relative abundance in heat-stressed and non-stressed samples (0.1460% and 0.0029%) (Table S2). Figure 4C illustrates the relative abundance of the most dominant orders in heat-stressed and non-heat-stressed samples as follows: *Clostridiales* (15.13% and 36.31%), *Enterobacteriales* (13.19% and 9.62%), *Lactobacillales* (37.02%, 37.35%), *Bacillales* (1.96% and 0.240%), *Streptophyta* (3.26% and 1.53%)*, Bacteroidales* (20.51% and 13.55%) and *Actinomycetales* (5.15% and 0.25%), respectively.

**Figure 5:**
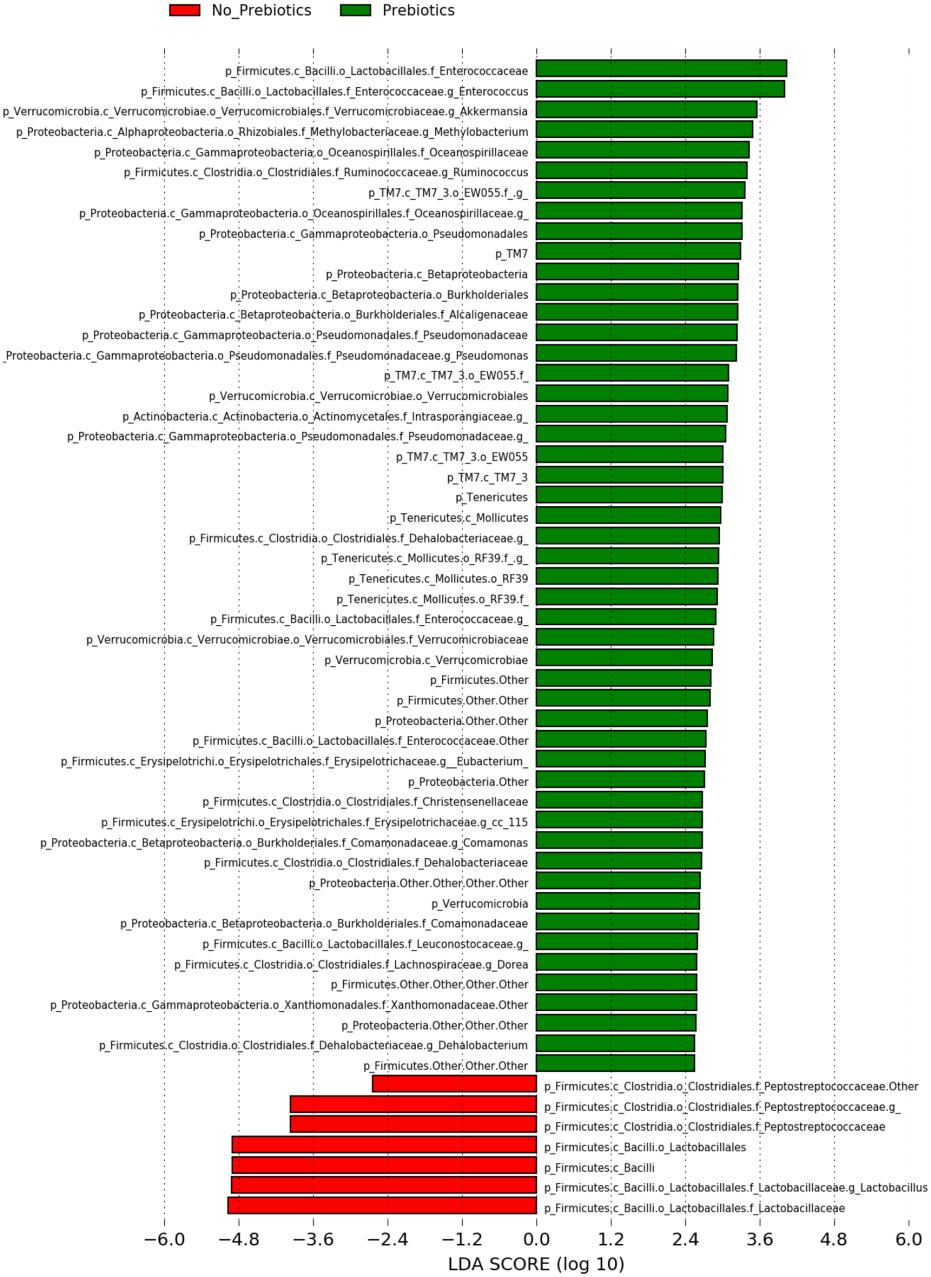
LEfSe core microbiome analysis. Data is presented as a histogram of the linear discriminative analysis (LDA) scores processed to feature the differentially rich taxa (core microbiome) between prebiotics and non-prebiotics samples.

**Figure 6:**
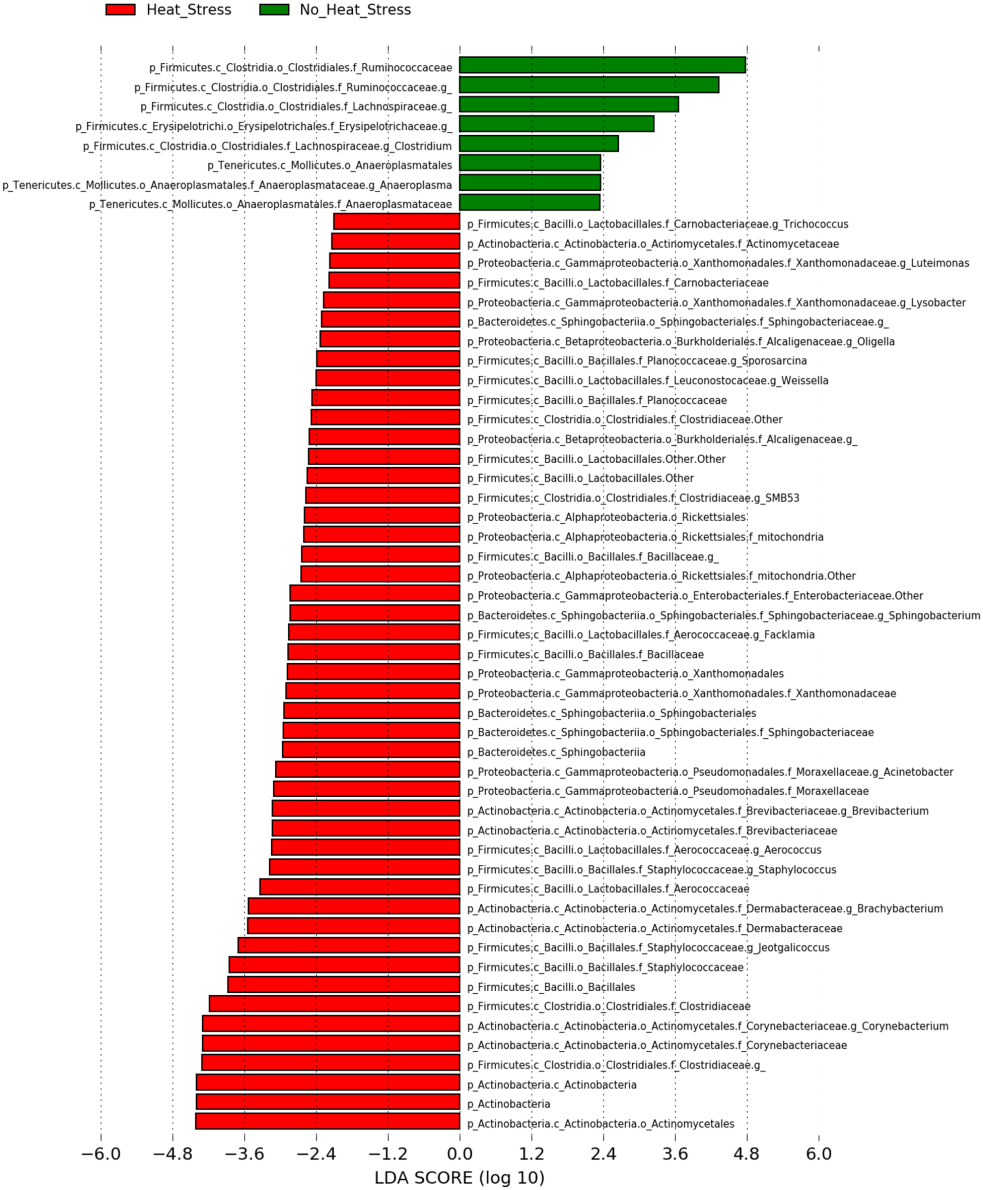
LEfSe core microbiome analysis. Data is presented as a histogram of the linear discriminative analysis (LDA) scores processed to feature differentially rich taxa (core microbiome) between heat-stressed and non-heat-stressed samples.

The LEfSe classification tool was used to characterize the differences between the core microbiome in the prebiotics sample and samples without prebiotics treatment (Fig. 5), in addition to the differences between the core microbiome in the heat-stressed and non-stressed samples at different levels (Fig. 6). The recorded results showed that the core microbiome in the prebiotics samples differed significantly from those samples without prebiotics treatment. The core microbiome in the prebiotics samples belonged to phyla *Proteobacteria and Firmicutes* including families *Erysipelotrichaceae*, *Xanthomonadaceae*, *Intrasporangiaceae* and *Oceanospirillaceae,* and genera *Eubacterium*, *Dorea*, *Methylobacterium*, *Pseudomonas*, *Dehalobacterium* and *Comamonas* (Fig. 5).

On the other hand, the LEfSe analysis showed that the core microbiome in the heat-stressed birds differed significantly from non-stressed ones (Fig. 6). The core microbiome in the heat-stressed bird included order *Lactobacillales*, families *Clostridiaceae*, *Corynebacteriaceae*, *Staphylococcaceae*, *Dermabacteriaceae*, *Brevibacteriaceae*, *Enterobacteriaceae*, *Bacillaceae*, *Aerococcaceae*, *Leuconostocaceae*, Aerococcaceae, *Xanthomonadaceae*, *Planococcaceae*, Alcaligenaceae, *Aerococcaceae*, *Carnobacteriaceae*, genus *Trichococcus*, *Aerococcus*, *Staphylococcus*, *Sporosarcina*, *Oligella*, *Luteimonas*, *Facklamia*, *Lysobacter*, *Brevibacterium, Corynebacterium*, *Jeotgalicoccus*, *Brachybacterium*, and *Weissella* (Fig. 6). The core microbiome of the non-heat stressed samples was affiliated to the families *Ruminococcaceae* and *Erysipelotrichaceae* (Fig. 6).

## Discussion

Concerns about how climate change would affect animal output are mounting. The average temperature is predicted to rise by 2 °C to 6 °C by the year 2100, posing a climatic challenge and increasing the likelihood of heat stress incidents in livestock species^22^. Our research demonstrated that chicken performance was negatively impacted by high ambient temperatures however, we proved that supplementing the birds with prebiotics reduced the effects of heat stress.

One of the major issues confronting the poultry business, particularly in tropical countries, is broiler breeding under high temperature. However, there is insufficient data on the negative effects of increased temperatures on the productivity output, body weight, gut microbiome, and immunological response in broilers as measured by antibody titer to different vaccines as well as total and differential leukocytic counts^4^.

In this study, exposure to HS revealed negative consequences on body weight, gut microbiome, and immunological response in broiler chickens. Results of the current study showed that HS (32–35 °C) had a negative impact on broiler BW, reduced appetite, lowered feed intake, and lowered final body weight. This declined growth of chickens bred under HS could be attributed to the fact that the elevated external temperature excited peripheral thermal sensors, which sent inhibitory nerve impulses to the hunger center in the hypothalamus, resulting in a reduction in the feed intake and increased water uptake to compensate for increased evaporative heat loss needs^14, 23^.

Our experiment showed that the (BW) in the first 2 weeks in the prebiotics groups was greater than those of the non-prebiotics groups. The overall FI masses in G1 and G2 were 3642 and 3389, while in G3 and G4 they were 3443 g and 3340, respectively. So, we concluded that prebiotic treatment maintains the feed capacity of birds while control groups show a severe decline in FI. In the 5th week (last week of our experiment), the bird performance in the groups administered prebiotics either under normal conditions (G3) or under HS (G4) showed improvement in BW and feed conversion ratio (FCR) in comparison with the other groups that didn’t receive any prebiotics either under normal conditions (G1) or under HS (G2). These results agree with those obtained by Sohail et al.^24^ who recorded that the addition of mannan-oligosaccharides and probiotics improved the performance of broiler chickens exposed to chronic HS. In addition, He et al.^25^ demonstrated that HS could cause weight loss in ducks and Luo et al.^26^ confirmed the same result in broiler chickens. These findings could be explained as the addition of prebiotics improves appetite, increases feed consumption, improves the gut microbiome, increases intestinal villi length, enhances nutrients absorption, and increases pathogen resistance that has a positive impact on the BW and lowers FCR^27^.

Interestingly, our prebiotics groups recorded decreased mortality rate even with heat stress in comparison to control groups, and this agrees with the study of Sugiharto who reported that prebiotics improved host defense and decreased avian mortality brought on by gut pathogen attack^12^.

Our results showed that the control group has a higher significant TWBC than the prebiotics group in weeks 4 and 5, and this result agrees with Amer et al.^28^ who reported that WBC levels of the control group were significantly higher than all immune treated in week 4 and 5. In addition, Al-Mansour et al.^29^ recorded that chicks fed yeast culture at a rate of 1.5 g/kg had significantly (*p* < 0.05) reduced leucocytic counts than controls. Also, we found that the groups exposed to HS recorded lower total white blood cells count (TWBC) which agrees with the findings of other studies^30^. These findings may be explained by the atrophy of all lymphoid tissues (bursa, thymus, spleen, or liver) since heat stress drastically decreased their weights. This may have happened as a result of the birds eating less feed, which would have provided them with fewer nutrients for their organs’ healthy growth^31^. According to Abo-Al-Ela et al.^32^, birds exposed to high ambient temperatures had a rise in plasma corticosterone, which in turn reduced the activity of their lymphoid tissues and their overall leucocyte count. However, G4 showed higher TWBC than G2 in week 4, and this agrees with Attia et al.^33^ who observed that administration of mannanoligosaccharides was accompanied by higher white blood cells (WBCs) than the other groups.

Regarding differential leukocytic count, monocyte showed a significant increase in prebiotics groups than the control group in weeks 2, 3, and 5. Our study agrees with Amer et al.^28^ who reported that beta glucan-treated groups show higher values of monocyte than other groups. Also, lymphocytes showed a significant increase in prebiotics groups than control in week 4, and this result matched with the other studies^28, 34, 35^. In addition to that the lymphocytes ratios were elevated in all groups in our study, and this is in accordance with Amer et al.^28^ who observed that lymphocytes represented the majority of total white blood cells in birds. This can be attributed to the vaccines given for all groups as these vaccines are viral types and the major reaction is cell-mediated immunity based. Therefore, it is logical to find a higher proportion of lymphocytes.

Monocytes showed a significant increase in the heat-stressed group when compared to the non-stressed groups and this finding agrees with that of Rocchi et al.^36^. It has been speculated that heat stress causes severe inflammation, especially in the gut resulting in increased intestinal permeability (leaky gut), damage of intestinal integrity due to the minimized expression of tight junction protein, and enhanced bacterial expansion^36^.

Average serum HI antibody titers against avian influenza (AI) and Newcastle disease (ND) vaccines indicated a significant increase in the prebiotics group than the control group in weeks 3 and 4, and this matched with previous studies^28, 37, 38^ which indicated that the β-1,3-d glucans molecules are powerful reticuloendothelial-modulators, whose immunobiological action is mediated via inducing the production of proinflammatory cytokines. Also, average serum HI antibody titers were significantly influenced by HS. It has been mentioned that HS causes oxidative stress, which lowers lymphoid organs’ weight and may be the primary factor in lower antibody titers for infectious illnesses like Al and ND. In addition, HS increases adrenal gland activity, which increases serum corticosteroid synthesis and inhibits interleukin-2 resulting in reduced titer of antibodies^39^. Meanwhile, birds administrated prebiotics in the drinking water (G3) showed a good immune response against the ND vaccine in comparison with the control group (G1) and the HS group (G2). Once again, this could be correlated to the overproduction of reactive oxygen species (ROS) in chickens exposed to environmental stressors like HS, which increases the production of free radicals that damage proteins and DNA through lipid peroxidation^40^. Prebiotics have been shown to boost the immune system, and the supplement’s ingredients, beta-d-glucan, and mannan-oligosaccharides, bind to the receptor mannose-specific type-1 fimbriae and stimulate gut immune cells to block pathogen colonization while favoring beneficial microbes like *Faecalibacterium*, which are frequently linked to gut health^12^.

Chicken droppings are considered the best choice of sampling to monitor the cecal microbiota because of their diversity, richness, and abundance. This enables researchers to collect samples without troubling the microbiota and to diminish stress on the chickens^41^. A greater understanding of the role of feed additives such as prebiotics on the microbial population and their diversity in response to HS could aid in the development of focused approaches to HS relief^2, 42^. High throughput sequencing was used to estimate the gut microbiota and their microecological composition in poultry^16, 43^.

Regarding the number of observed bacterial microbiota species within samples in our study, birds showed higher diversity in week 3 than week 5. This may be as a result of the chickens being under stress in the last week of rearing due to the sudden increase in their body weights. In week 1 the species’ richness begins to grow and propagate. High ambient temperature is an important environmental indicator that regulates bacterial expansion and progression^44^. Continuously elevated temperatures can lead to functional problems, such as intestinal dysbiosis^44, 45^.

In this study, there was a significant difference recorded between control groups and prebiotics-treated groups in terms of observed species richness. Prebiotics-treated groups showed higher diversity than the control groups as prebiotics increased diversity. HS resulted in a significant decrease in population, but the prebiotics could compensate for the effect of HS and maintain its bacterial population.

The rarefaction curves and alpha diversity parameters were evaluated, and there was a significant difference in the alpha diversity indices, showing that HS had a major effect on gut microbiota diversity. The data revealed that the chicken gut microbiome was mainly comprised of five phyla: *Firmicutes*, *Proteobacteria*, *Bacteroidetes, Actinobacteria*, and *Cyanobacteria*. *Firmicutes* was the major microbial group, accounting for more than 45% of the microbiome. In addition, we found *Tenericutes, Verrucomicrobia* and *Acidobacteria* in lower concentrations (less than 0.2%) in the broilers’ gut. Our study agrees with the study of Wen et al.^46^ who found that *Firmicutes* accounted for more than 60% of the chicken gut microbiota.

In the current study, in week 5 under heat stress, the *Firmicutes* level was higher in the control group than in the prebiotics groups while *Bacteroidetes* were higher in prebiotics groups than in the control groups under heat stress applications. Adding prebiotics to the drinking water of birds increases the count of beneficial microbiome especially *Lactobacillus* which improve performance and plays an important function in food fermentation^47^. *Lactobacillus dominance* in the intestine has been linked to resistance to pathogens and illnesses^48^. Our data revealed that *L. murinus*, *L. ruminus*, and *L. casei* were the most abundant *lactobacilli* in our prebiotics-treated samples*. Lactobacillus* has been found to be extremely abundant in the distal gut and its deficiency is usually linked to the disease conditions^49, 50^.

*Bacteroidetes* are crucial complex carbohydrate degraders and short-chain fatty acid (SCFA) makers that are well-suited to the distal gut and serve as significant commensals in the chicken gut^51, 52^. *Firmicutes* primarily break down oligosaccharides or convert lactate or acetate, two metabolic waste products, into butyrate^53^. The *Firmicutes*-to-*Bacteroidetes* ratio was considerably higher when more *Firmicutes* and fewer *Bacteroidetes* were found in the gut microbiota of stressed broilers^54^. In our study *Firmicutes*-to-*Bacteroidetes* in control and prebiotics groups was 4.08 and 3.07, respectively.

*Proteobacteria* have been found to grow as a result of antibiotic use or inflammation in both people and animals, and this phenomenon is thought to be a microbial indicator of gut disturbance and epithelial malfunction^55, 56^. In our investigation, the total percentage of *Proteobacteria* in the control groups (38.3%) was higher than that in the prebiotics groups (26.7%), this strongly proves the added value of using prebiotics to improve the avian gut microbiome.

Animal-developed *Enterobacteriaceae* bacteria such as *Klebsiella pneumoniae*, *Escherichia coli*, *Proteus mirabilis*, and *Salmonella* spp. especially serotypes *Salmonella Pullorum* and *Salmonella Gallinarum* are significant food-borne zoonotic bacteria that are prevalent in the broiler breeding sector^57, 58^. In our results, the overall prevalence of *Enterobacteriaceae* was 36.75% in control groups and 23.19% in prebiotics groups. Moreover, during the last week under heat stress exposure, the prevalence of *Enterobacteriaceae* in control is higher than in prebiotics groups. This again explored the added value of using prebiotics to improve the avian gut microbiome and alleviate the effect of heat stress.

In our experiment genus *Brevibacterium*, and *Facklamia* showed a significant increase in the heat-stressed group than the non-heat-stressed group. Other research indicated that few *Brevibacterium-*like microbes isolated from infected chicken were obtained from aspirates taken during necropsy causing bumblefoot (granuloma) lesions, Moreover, some species-level identification was not established^59^. *Facklamia* has been reported to produce septicemia and meningitis, both of which can be fatal^60^. This reflects the negative impact of heat stress on the avian gut microbiome and valorizes the positive impact of using prebiotics to alleviate the effect of heat stress as the abundance of these pathogens was either eradicated or significantly decreased in the prebiotics-treated groups.

## Conclusion

The study of microbial structure and its interaction is useful for discovering the effect of high temperature on the intestinal barrier and can provide a hypothetical foundation and experimental suggestions for restoring body destruction affected by heat stress. In our experiments, the birds’ exposure to heat stress reflected badly on the bird performance and reduced bird immunity. Meanwhile, the addition of prebiotics in the drinking water during the rearing period of broiler chickens alleviated the negative impact of heat stress. Prebiotics were able to improve bird appetite, increase feed intake, lower FCR, improve the immunological parameters of the bird, boost the bird immune response against infections, and increase the effectiveness of bird immune response to vaccines. Moreover, using prebiotics improved the birds’ gut microbiome and alleviated the negative effect of heat stress. Administering prebiotics significantly increased the relative abundance of beneficial bacteria and eradicated pathogenic ones in the chicken gut microbiome.

### Supplementary Information

Supplementary Table S1.

Supplementary Table S2.

## Data Availability

16S sequence data was submitted to the Sequence Read Archive (SRA) under Bioproject (PRJNA977599) and SRA study (SRR24784609-15). The data was assigned Accession Numbers SAMN35526087, SAMN35526088, SAMN35526089, SAMN35526090, SAMN35526091, SAMN35526092 and SAMN35526093. The data has been released and it is available via the following link: https://submit.ncbi.nlm.nih.gov/subs/bioproject/SUB13482173/overview.
